# Association of Systemic Comorbidities with Dry Eye Disease

**DOI:** 10.3390/jcm9072040

**Published:** 2020-06-29

**Authors:** Motoko Kawashima, Masakazu Yamada, Chika Shigeyasu, Kazuhisa Suwaki, Miki Uchino, Yoshimune Hiratsuka, Norihiko Yokoi, Kazuo Tsubota, for the DECS-J Study Group

**Affiliations:** 1 Department of Ophthalmology, Keio University School of Medicine, Tokyo 1608582, Japan; uchinomiki@yahoo.co.jp (M.U.); tsubota@z3.keio.jp (K.T.); 2 Department of Ophthalmology, Kyorin University School of Medicine, Tokyo 1818611, Japan; yamadamasakazu@ks.kyorin-u.ac.jp (M.Y.); shigeyasu@eye-center.org (C.S.); 3 Department of Japan Medical Affairs, Santen Pharmaceutical Co., Ltd., Osaka 5308552, Japan; kazuhisa.suwaki@santen.com; 4 Department of Ophthalmology, Juntendo University Graduate School of Medicine, Tokyo 1138431, Japan; yoshi-h@tkf.att.ne.jp; 5 Department of Ophthalmology, Kyoto Prefectural University of Medicine, Kyoto 6020841, Japan; nyokoi@koto.kpu-m.ac.jp

**Keywords:** cross-sectional study, dry eye disease, systemic comorbidity

## Abstract

We investigated the association between dry eye disease and systemic comorbidities, including dry eye subtype, quality of life (QOL) and health utility among patients with dry eye disease. This cross-sectional, observational study enrolled 449 patients with dry eye disease (386 females; mean age, 62.6 ± 15.7 [range, 21–90] years). Ophthalmic examination findings included tear film break-up time (TBUT), Schirmer I value and keratoconjunctival staining score. QOL and health utility were evaluated using the Dry Eye-Related Quality-of-Life Score (DEQS) and Human Utility Index Mark 3 (HUI-3), respectively. Background information, including systemic comorbidities, was obtained. Prevalence of systemic comorbidities was 48.8% (219/449). No significant difference occurred between DEQS and systemic comorbidity. However, patients with dry eye disease and systemic comorbidities (depression and insomnia) exhibited significantly worse ocular surface parameters, particularly regarding TBUT, than those without. Dry eye disease with insomnia or depression comorbidity significantly correlated with friction-related diseases (including conjunctivochalasis or lid wiper epitheliopathy). A high prevalence of several systemic comorbidities occurred in patients with dry eye disease. This study shows an association between ocular signs and systemic comorbidities, particularly depression and insomnia. Ophthalmologists should be aware of patients’ systemic comorbidities in the diagnosis and management of dry eye disease.

## 1. Introduction

Dry eye disease is a common disease caused by numerous factors including ocular surface problems, environmental factors such as humidity and wind, and systemic conditions. In addition, a variety of systemic medications can also induce dry eye disease [1,2,3,4]. Although it is widely recognized as an ocular surface disease, associated systemic conditions are not negligible, as they can negatively impact ocular health. Such systemic conditions include rheumatoid arthritis, vitamin A deficiency, bone marrow transplantation and postmenopausal estrogen therapy. Besides these causal relationships, epidemiological studies have also reported the association of dry eye disease with systemic diseases, including ischemic heart disease and hyperlipidemia. Depression, antidepressant use and insomnia are also reported to be associated with dry eye disease. Schein et al. reported that elderly individuals with systemic comorbidities are likely to have symptoms of dry eye disease [5]. These findings from statistical approaches and epidemiological studies should be carefully interpreted, as dry eye disease is a common disease and the elderly tend to be affected by multiple comorbidities.

Previous studies have demonstrated aging as the most clearly documented risk factor for dry eye disease [6]. Accordingly, the prevalence of this disease is increasing particularly in modern age societies like Japan. However, most previous studies reporting on the prevalence of dry eye disease and systemic comorbidities [4,7,8] have been based on epidemiological questionnaires. Therefore, the relationship between ocular parameters and systemic comorbidities has not been well investigated. For example, a previous study by Wang et al. revealed that a number of systemic diseases correlate with dry eye disease, but since the authors did not include any ocular examination, the relationship of specific dry eye parameters to these other conditions was not determined [9]. Therefore, we investigated the association between dry eye disease and systemic comorbidities, as part of the clinic-based Dry Eye Cross-Sectional Study (DECS-J) in Japan.

## 2. Materials and Methods

### 2.1. Ethics

This study was conducted in accordance with the guidelines of the World Medical Association, Declaration of Helsinki and the Ethical Guidelines for Medical and Health Research Involving Human Subjects in Japan. Subjects received full explanation of the procedures and provided written informed consent before enrollment. The study protocol was approved by the Institutional Review Board of the Clinical Study, Ryogoku Eye Clinic, Tokyo, Japan. The study was registered in a public registration system (University Hospital Medical Information Network registry no. UMIN 000015890) [10].

### 2.2. Study Population

We collected data from the DECS-J, which was an observational study conducted in 10 eye clinics in Japan. All investigators were specialists in ocular surface disorders and dry eye disease and belonged to the Japanese Dry Eye Society. To ensure the quality of the survey, two investigators’ meetings were held prior to the start of patient enrollment to discuss the study protocols and examination procedures.

We consecutively enrolled outpatients who were at least 20 years old and were newly or previously diagnosed with dry eye disease. The criteria for diagnosis was as follows: (1) at least one abnormal tear examination result [Schirmer I test ≤ 5 mm, tear film break-up time (TBUT) ≤ 5 s]; (2) abnormal results in ocular surface vital staining tests (fluorescein keratoconjunctival staining score ≥ 3); and (3) presence of dry eye symptoms [11]. Subjects who met two or all three criteria were considered to probably or definitely have dry eye disease, respectively, and were included in the study. Up to 50 patients were enrolled at each of the 10 study sites from 1 December 2014 to 28 February 2015.

A total of 449 patients with an average age of 62.6 ± 15.7 years were included in this study. Of these, 386 (86.0%) were females. Table 1 shows the age distribution of the patients in this study.

### 2.3. Systemic Comorbidities and Oral Medicine

Patients were asked to provide information regarding systemic comorbidities and oral medicine. Hypertension, insomnia and depression were defined as having hypertension or taking an antihypertensive medication; as having insomnia or taking a sedative; and as having depression or taking an antidepressant, respectively.

### 2.4. Quality of Life (QOL) and Health Utility Assessment

QOL and health utility among patients with dry eye disease were evaluated using the DEQS [12] and the Human Utility Index Mark 3 (HUI-3) questionnaires [13], respectively. The DEQS consists of 15 questions and is scored using an overall summary scale. The score derived from this questionnaire is considered to be a quantitative measure of dry eye symptoms, in which 0 indicates the best and 100 indicates the worst. Test/retest reliability and discriminant validity of the DEQS were confirmed in a previous study, showing that the score was significantly higher in patients with dry eye disease than in healthy controls (33.7 vs. 6.0) [9].

The HUI-3 is a standard method for assessing the utility value. It is a multi-attribute utility classification system for preferences associated with generic health states. It consists of 15 questions assessing eight health attributes: vision, hearing, speech, ambulation, dexterity, emotion, cognition and pain. Individual sets of answers were converted to utility values, using the method described by Furlong et al. [14]. A utility value representing the overall health state was derived by applying a weighted scoring algorithm, in which utility is defined on a continuous scale from 0 to 1, where 0 corresponds to the worst possible QOL outcome (equal to death) and 1 corresponds to the best possible one (equal to perfect health). Patients were asked to fill out the questionnaires at home and return them by post.

### 2.5. Ophthalmic Evaluation

Ophthalmic examinations included assessment of conjunctival and corneal vital staining with fluorescein sodium, measurement of the TBUT and the Schirmer I test.

Test strips containing fluorescein sodium (Fluores Ocular Examination Test Paper, Ayumi Pharmaceutical Co., Tokyo, Japan) were used for vital staining and TBUT measurement. After the tip of a wet test strip had been applied to the inferior temporal tear meniscus, patients were asked to blink several times to ensure adequate mixing of the fluorescein dye with the tears. The time interval between the last complete blink and the appearance of the first corneal dark spot was timed using a stopwatch. The average of three measurements was recorded as the TBUT. Corneal and conjunctival epithelial damage was then evaluated via corneal fluorescein staining, according to the National Eye Institute grading system [15]. Briefly, corneal staining was graded with a score of 0–3, assigned to each of five corneal zones (superior, nasal, central, inferior and temporal), with a maximum total score of 15. The fluorescein staining score of the keratoconjunctival was determined according to the modified grading system of van Bijsterveld [16], according to which each eye is divided into three sections (temporal conjunctiva, cornea and nasal conjunctiva) and scored from 0 to 3. The final score ranged from 0 (minimum) to 9 (maximum). The Schirmer I test was performed without topical anesthesia after all other examinations had been completed. A Schirmer test strip (Ayumi Pharmaceutical Co.) was placed on the outer one third of the temporal lower conjunctival fornix for 5 min. The strip was then removed, and the length of the wet filter paper was recorded in millimeters. To avoid any effect of keratoconjunctival staining on the Schirmer I test, the tests were performed 15 min apart, at minimum.

For each patient, the eye that met the most criteria for dry eye diagnosis was included in the study. If both eyes met the same number of criteria, the eye with the higher fluorescein staining score and the shorter TBUT was included. If these values were the same for both eyes, the right eye was used.

### 2.6. Classification of Dry Eye Subgroups

#### 2.6.1. Aqueous-Deficient (AD)/sTBUT

We classified the subjects into aqueous-deficient (AD) and short TBUT dry eye subgroups. The AD dry eye group was comprised of subjects who fulfilled the following criteria: (1) the presence of dry eye symptoms and (2) decreased tear production (Schirmer value ≤ 5 mm). The short TBUT dry eye subgroup included subjects who met the following conditions: (1) the presence of dry eye symptoms, (2) abnormal tear stability (TBUT ≤ 5 s), (3) no abnormality in tear production (Schirmer value > 5 mm), and (4) no abnormality in the ocular surface vital staining test (keratoconjunctival score < 3) [17].

#### 2.6.2. Meibomian Gland Function (MGD+/−)

We evaluated meibomian gland function (MGD in the central one-third of the upper eyelid using slit-lamp biomicroscopy. The patient was instructed to look down while the investigator gently and partially everted the upper lid to examine the lid margin. Typical pictures of the following signs/findings were distributed to each investigator to aid in the examination: (1) one or more abnormal findings around the meibomian gland orifices, such as vascular engorgement, anterior or posterior displacement of the mucocutaneous junction or irregularity of the lid margin (2) orifice obstruction, such as plugging, pouting and ridges [18]; and (3) hypersecretion or hyposecretion of the meibum [19]. We diagnosed MGD when all the signs and findings listed in the criteria were present [17].

#### 2.6.3. Friction-Related Diseases (FRD+/−)

We considered that subjects had friction-related conditions when any of the following conditions were present: lid wiper epitheliopathy (in the upper and lower lids), conjunctivochalasis (at the central site of the lower eyelid) or superior limbic keratoconjunctivitis [17,20,21,22].

### 2.7. Statistical Methods

We used one eye from each subject for the analysis, as explained above (Section 2.5). Data for all parameters are presented as the mean ± standard deviation. We performed all statistical analyses using SAS software, version 9.4 (SAS Inc., Cary, NC, USA). For comparisons between groups, we used Fisher’s exact test for dichotomous variables and Student’s *t*-test for continuous variables. We performed a linear model analysis to adjust for the effects of age. To estimate the association between a given independent variable and the outcome after adjustment for age, multiple linear regression was used for continuous variables and multiple logistic regression was used for binary outcomes. We considered a *p*-value lower than 0.05 as statistically significant for all analyses.

## 3. Results

The prevalence of systemic comorbidities was 48.8% (219/449 cases). The most prevalent comorbidity was hypertension in 108 cases (24.1%), followed by insomnia in 67 cases (14.9%) and depression in 20 cases (4.5%). The 113 cases categorized as “others” included hyperlipidemia (15), Sjogren’s syndrome (7), abnormal thyroid function (7), hypercholesteremia (7), angina (4), arrhythmia (4) and so on. Ninety-eight patients (21.8%) were taking anti-hypertensives and 55 (12.2%) were taking sedatives (Table 2).

Patients with dry eye disease who had any systemic comorbidities exhibited a significantly lower Schirmer value (*p* = 0.004), shorter TBUT (*p* = 0.002), higher keratoconjunctival staining score (*p* = 0.0002) and lower utility value estimated by HUI-3 (*p* = 0.003) than those without systemic comorbidities (Table 3). After adjustment for age, differences in TBUT, keratoconjunctival staining score and HUI-3 utility value remained statistically significant (*p* = 0.011, *p* < 0.001 and *p* = 0.03, respectively). Patients who in addition to dry eye disease also had hypertension or depression exhibited a significantly lower Schirmer value than those without (*p* = 0.008 and *p* = 0.04, respectively), while those who had insomnia or depression exhibited a significantly shorter TBUT than those without (*p* = 0.01 and *p* = 0.04, respectively). The DEQS score was lower in patients with dry eye disease and hypertension (*p* = 0.003). After adjustment for age, the TBUT in patients with depression or insomnia remained significantly shorter (*p* = 0.04 and *p* = 0.04, respectively).

Regarding oral medicine use, patients with dry eye disease who were prescribed antidepressants had a significantly lower Schirmer value (*p* = 0.04) and lower TBUT (*p* = 0.04), while those who used anti-hypertensives had a significantly lower Schirmer value (*p* = 0.009) and lower DEQS score (*p* = 0.03) than those who did not receive such medications. After adjustment for age, antidepressant use remained significantly different for both the Schirmer value and TBUT (*p* = 0.05 and *p* = 0.04, respectively; Table 4).

We examined the relationship between dry eye disease subtypes and systemic comorbidities. We found that patients with hypertension and insomnia had a significantly higher prevalence of MGD (hypertension: odds ratio [OR] 2.65, 95% confidence interval [CI] 1.64–4.28, *p* < 0.01; insomnia: OR 1.94, 95% CI 1.10–3.42, *p* = 0.02). Patients with dry eye disease and hypertension, insomnia or depression had a significantly higher prevalence of FRD (hypertension: OR 1.56, 95% CI 1.00–2.44, *p* = 0.05; insomnia: OR 2.66, 95% CI 1.57–4.51, *p* < 0.01; depression: OR 4.92, 95% CI 1.85–13.08, *p* < 0.01). After the OR was adjusted for age, insomnia and depression, comorbidities with dry eye disease were significantly associated with FRD (insomnia: OR 2.16, 95% CI 1.26–3.72, *p* < 0.01; depression: OR 4.89, 95% CI 1.81–13.22, *p* < 0.01; Table 5).

## 4. Discussion

In this study, we investigated the relationship between the severity of dry eye disease and systemic comorbidities. Aging is the most clearly documented risk factor for dry eye disease [6], and the elderly tend to be affected by multiple comorbidities. As expected, the average age of subjects in our study was relatively high (62.6 years on average), and approximately half of the patients had systemic comorbidities. Prevalent systemic comorbidities were hypertension, insomnia and depression. We found that patients with dry eye disease and any systemic comorbidity exhibited significantly worse utility, as estimated by HUI-3, than those without. In addition, those with systemic comorbidities had a higher severity of dry eye disease, as demonstrated by objective parameters, such as the TBUT and Schirmer value. These correlations remained statistically significant after adjustment for age. Schein et al. reported that the presence of many systemic comorbidities is strongly associated with reported symptoms of dry eye disease [5], although no association was seen between systemic comorbidity and Schirmer or Rose Bengal test scores. Our findings suggest that the presence of systemic comorbidities may compromise the ocular surface, as well as the QOL of patients with dry eye disease. However, systemic comorbidities include numerous conditions with different pathophysiology, which affect various parts of the body. Our findings are based on a statistical and epidemiological approach and therefore must be re-examined cautiously in further studies.

In this study, we found that patients with dry eye disease and depression showed a significantly shorter TBUT after adjustment for age. Interestingly, those taking antidepressants showed a significantly lower Schirmer value, in addition to a shorter TBUT, after such adjustments. These results suggest that depression and/or anti-depressant use is associated with poor tear film stability, and that antidepressant use may worsen tear secretion in addition to tear stability, which is an indicator of dry eye severity. Previous studies, mostly involving epidemiological questionnaires, have also reported a relationship between depression and dry eye disease [23,24,25,26]. In a comparison of depressive and control groups, one case-control study revealed significantly lower Schirmer scores and shorter TBUT, as well as a consistently higher Oxford scores in individuals with depression [27]. Similarly, we demonstrated an association between depression/anti-depressant use and dry eye severity by undertaking objective measurements, although the mechanisms involved in this association remain unknown.

Another interesting finding in our study is that patients with dry eye disease and insomnia showed a significantly shorter TBUT after adjustment for age. Several investigators have recently reported the association of sleep disturbances, like insomnia, and dry eye disease [28,29,30]. Our results also support such an association. Insomnia is a common problem in the elderly and sedative drugs, including benzodiazepines, are frequently prescribed for this condition in Japan. The mechanism involved in the association between insomnia and dry eye disease may be derived from insomnia itself. Alternatively, the intake of sedative drugs may have some influence. Further studies are needed to clarify this issue.

In addition, we found that FRD was significantly associated with dry eye disease and was a comorbidity with insomnia or depression, even after adjusting for age. The presence of FRD, including conjunctivochalasis and lid wiper epitheliopathy, is a predisposing factor for dry eye disease and its severity. To our knowledge, an association between FRD and depression or insomnia has not yet been reported. We also noted that antidepressant users had decreased tear secretions. Since FRDs are associated with lower tear volume and quality (e.g., secreted mucin), dry eye disease is more likely to be associated with FRD when depression and insomnia are also present than when they are not. Another possible explanation is that anti-depressant and sedative drugs may induce blepharospasm [31,32], as blinking problems induced by drugs may result in the development of FRDs. Although the cause is not known, our study suggests that anti-depressant use may reduce tear secretion and worsen ocular surface conditions.

In this study, we demonstrated that depression and insomnia comorbidity with dry eye disease increased dry eye severity and that, in turn, ocular symptoms may worsen depression/insomnia, thus creating a vicious circle. Therefore, ophthalmologists should be aware of patients’ systemic comorbidities in the diagnosis and treatment of dry eye disease. Similarly, psychologists should be aware of patients’ eye conditions when considering mental health management. We feel that it is especially important for psychiatrists to take this into account, particularly when prescribing antidepressants, which may aggravate dry eye signs.

Our findings must be considered along with several limitations. First, detailed patient medical information could not be collected regarding the severity of depression, details of medications, duration of systemic comorbidities and duration of oral medicine use. This information is important for establishing the underlying mechanisms of dry eye severity and systemic comorbidities. Dry eye disease is common in patients with depression, especially those of older age who have been experiencing depression and taking antidepressant medication for longer periods. Therefore, future studies should focus on patients with depression and dry eye disease. Second, our study was based on a cross-sectional design; therefore, it is difficult to ascertain whether the observed associations are due to causal effects. Third, we included patients with dry eye disease who had been receiving various treatments. Therefore, there may have been differences in the clinical presentation of these patients.

In conclusion, in the clinical setting ophthalmologists should pay careful attention to the systemic comorbidities of patients with dry eye disease, and especially of older adults who have a higher prevalence of such comorbidities. Comorbidities including depression, insomnia and the use of antidepressants are associated with dry eye disease severity.

## Figures and Tables

**Table 1:** Age and sex distribution of the patients in this study.

Age (Years)	Sex		Total (*n* (%))
	Male (*n* (%))	Female (*n* (%))	
<40	7 (11.11)	37 (9.59)	44 (9.8)
40–64	21 (33.33)	139 (36.01)	160 (35.63)
≥65	35 (55.56)	210 (54.4)	245 (54.57)
Total	63 (100)	386 (100)	449 (100)

**Table 2:** Prevalence of systemic comorbidities and oral medicine use.

		Cases	%	Age (Average ± SD)	Female (%)	Male (%)
Total		449		62.6 ± 15.6	386 (86.0)	63 (14.0)
Systemic comorbidity *1	(−)	230	51.2	56.3 ± 16.1	195 (84.8)	35 (15.2)
	(+)	219	48.8	69.2 ± 12.1	191 (87.2)	28 (12.8)
Details *2	Hypertension	108	24.1	73.1 ± 10.8	91 (84.3)	17 (15.7)
	Insomnia	67	14.9	71.0 ± 11.3	59 (88.1)	8 (11.9)
	Depression	20	4.5	64.9 ± 13.5	19 (95.0)	1 (5.0)
	DM	16	3.6	70.6 ± 11.6	14 (87.5)	2 (12.5)
	RA	15	3.3	70.4 ± 8.4	15 (100.0)	0 (0.0)
	Others	113	25.2	68.1 ± 12.4	105 (92.9)	8 (7.1)
Oral medicine *2	Antihypertensive	98	21.8	73.2 ± 11.1	82 (83.7)	16 (16.3)
	Sedative	55	12.2	70.9 ± 11.1	48 (87.3)	7 (12.7)
	Antidepressant	20	4.5	64.9 ± 13.5	19 (95.0)	1 (5.0)
	Others	147	32.7	68.5 ± 12.4	134 (91.2)	13 (8.8)

DM, diabetes mellitus; RA, rheumatoid arthritis; SD, standard deviation. *1: (+) indicates more than one comorbidity selected among hypertension, insomnia, depression, DM, RA or others. *2: multiple answers allowed.

**Table 3:** Systemic comorbidities and dry eye parameters.

		No. of Cases	Schirmer Test (mm) (Mean ± SD)	TBUT (s) (Mean ± SD)	Keratoconjunctival Staining Score (Points) (Mean ± SD)	DEQS (Mean ± SD)	HUI (Mean ± SD)
Systemic comorbidity	+	219	8.38 ± 8.20	2.49 ± 1.59	3.16 ± 1.86	25.15 ± 20.69	0.72 ± 0.23
	−	230	10.98 ± 10.39	2.94 ± 1.51	2.47 ± 1.99	28.35 ± 20.50	0.79 ± 0.20
	*p*-value		0.004 *	0.002 *	0.0002 *	0.104	0.003 *
	Age-adjusted *p*-value		0.656	0.011 *	<0.001 *	0.411	0.030 *
Hypertension *	+	108	7.61 ± 7.19	2.66 ± 1.65	2.96 ± 1.75	21.71 ± 18.68	0.73 ± 0.24
	−	341	10.36 ± 9.99	2.74 ± 1.54	2.75 ± 2.02	28.40 ± 20.98	0.76 ± 0.21
	*p*-value		0.008	0.645	0.334	0.004 *	0.275
	Age-adjusted *p*-value		0.694	0.820	0.347	0.406	0.897
Insomnia *	+	67	8.63 ± 8.96	2.31 ± 1.55	2.81 ± 1.80	26.31 ± 19.59	0.74 ± 0.19
	−	382	9.88 ± 9.53	2.80 ± 1.56	2.80 ± 1.99	26.87 ± 20.83	0.76 ± 0.22
	*p*-value		0.318	0.018	0.993	0.84	0.516
	Age-adjusted *p*-value		0.698	0.044 *	0.960	0.249	0.938
Depression *	+	20	5.50 ± 5.85	2.03 ± 1.84	2.15 ± 1.90	26.05 ± 18.05	0.725 ± 0.213
	−	429	9.89 ± 9.54	2.76 ± 1.55	2.83 ± 1.96	26.82 ± 20.76	0.756 ± 0.215
	*p*-value		0.042 *	0.043 *	0.127	0.874	0.55
	Age-adjusted *p*-value		0.056	0.049 *	0.125	0.993	0.598

TBUT, tear film break up time; DEQS, Dry Eye-Related Quality-of-Life Score; HUI, health utilities index; * *p* ≤ 0.05; “+”: presence; “−”: absence.

**Table 4:** Oral medicine and dry eye parameters.

		No. of Cases	Schirmer Value (mm)	TBUT (s)	Fluorescein Staining Score	DEQS	HUI
Antihypertensive	(+)	98	7.50 ± 6.84	2.74 ± 1.68	2.92 ± 1.73	21.39 ± 18.95	0.73 ± 0.25
	(−)	351	10.31 ± 9.99	2.72 ± 1.53	2.77 ± 2.02	28.33 ± 20.86	0.76 ± 0.20
	*p*		0.009 *	0.901	0.514	0.003 *	0.272
	Age-adjusted		0.626	0.413	0.548	0.342	0.853
Sedative	(+)	55	8.64 ± 9.13	2.38 ± 1.61	2.93 ± 1.82	27.54 ± 20.16	0.74 ± 0.20
	(−)	394	9.84 ± 9.49	2.77 ± 1.55	2.79 ± 1.98	26.69 ± 20.72	0.76 ± 0.22
	*p*		0.378	0.078	0.619	0.779	0.485
	Age-adjusted		0.741	0.149	0.649	0.138	0.837
Antidepressant	(+)	20	5.50 ± 5.85	2.03 ± 1.84	2.15 ± 1.90	26.05 ± 18.05	0.73 ± 0.21
	(−)	429	9.89 ± 9.54	2.76 ± 1.55	2.83 ± 1.96	26.82 ± 20.76	0.76 ± 0.22
	*p*		0.042 *	0.043 *	0.127	0.874	0.55
	Age-adjusted		0.056	0.049 *	0.125	0.993	0.598

TBUT: tear film break up time; DEQS: Dry Eye-Related Quality-of-Life Score; HUI: Health Utilities Index. * *p* < 0.05. Mean ± SD; “+”: presence; “−”: absence.

**Table 5:** Association between dry eye subtype and systemic comorbidities.

DE Subtype (ADDE/sTBUT)				
	OR (95% Cl)	*p* Value	Adjusted OR (95% CI)	*p* Value
Hypertension	1.46 (0.82–2.58)	0.20	1.17 (0.64–2.15)	0.61
Insomnia	0.97 (0.51–1.81)	0.92	0.80 (0.41–1.53)	0.49
Depression	1.69 (0.54–5.27)	0.36	1.67 (0.53–5.22)	0.38
**MGD (Yes/No)**				
	**OR (95% CI)**	***p* Value**	**Adjusted OR 95% CI)**	***p* Value**
Hypertension	2.65 (1.64–4.28)	<0.01	1.33 (0.78–2.27)	0.30
Insomnia	1.94 (1.10–3.42)	0.02	1.21 (0.66–2.21)	0.54
Depression	0.38 (0.09–1.67)	0.20	0.33 (0.07–1.49)	0.15
**FRD (Yes/No)**				
	**OR (95% CI)**	***p* Value**	**Adjusted OR (95% CI)**	***p* Value**
Hypertension	1.56 (1.00–2.44)	0.05	1.10 (0.68–1.79)	0.70
Insomnia	2.66 (1.57–4.51)	<0.01	2.16 (1.26–3.72)	<0.01
Depression	4.92 (1.85–13.08)	<0.01	4.89 (1.81–13.22)	<0.01

OR, odds ratio; CI, confidence Interval; ADDE, aqueous-deficient dry eye; DE, dry eye; sTBUT, short tear film break up time dry eye; MGD, meibomian gland dysfunction; FRD, friction-related disease.
